# The role of RNA modification in the generation of acquired drug resistance in glioma

**DOI:** 10.3389/fgene.2022.1032286

**Published:** 2022-11-11

**Authors:** Yu Yan, Wei Wei, Shengrong Long, Shengda Ye, Bin Yang, Jiazhi Jiang, Xiang Li, Jincao Chen

**Affiliations:** ^1^ Department of Neurosurgery, Zhongnan Hospital, Wuhan University, Wuhan, China; ^2^ Brain Research Center, Zhongnan Hospital, Wuhan University, Wuhan, China

**Keywords:** epigenetic regulation, RNA modification, glioblastoma, multi-omics interactions, m6A, m5C, tumor-acquired drug resistance

## Abstract

Glioma is the most common malignant tumor in the central nervous system. The clinical treatment strategy is mainly surgery combined with concurrent temozolomide chemotherapy, but patients can develop drug resistance during treatment, which severely limits its therapeutic efficacy. Epigenetic regulation at the RNA level is plastic and adaptable, and it can induce a variety of tumor responses to drugs. The regulators of RNA modification include methyltransferases, demethylases, and methylation binding proteins; these are also considered to play an important role in the development, prognosis, and therapeutic response of gliomas, which provides a basis for finding new targets of epigenetic drugs and resetting the sensitivity of tumor cells to temozolomide. This review discusses the relationship between the development of adaptive drug resistance and RNA modification in glioma and summarizes the progress of several major RNA modification strategies in this field, especially RNA m6A modification, m5C modification, and adenosine-to-inosine editing.

## 1 Introduction

Gliomas are the most common primary central nervous system tumors and similar histological features to normal glial cells (i.e, astrocytes, oligodendrocytes, and ependymal cells), accounting for approximately 80% of malignant tumors of the central nervous system (Bauchet and Ostrom, 2019). According to the 2021 World Health Organization classification, glioblastoma (GBM) is defined as IDH-wildtype and assigned a grade 4 classification with high malignant potential, a short survival period, and poor reactions to treatment. GBM mostly occurs in the supratentorial area of the brain. The average age of patients with GBM in developed countries is 64 years old, and they show rapid progression and poor prognosis. The median overall survival time is 16–18 months, and the 5-year survival rate after diagnosis is 5.8% (Alexander and Cloughesy, 2017; Wang et al., 2021a; Ostrom et al., 2021).

In the clinical setting, the standard treatment for patients with primary GBM is surgical resection with a maximum safety margin, postoperative radiotherapy combined with concurrent temozolomide (TMZ) chemotherapy, and then 6-week of TMZ maintenance therapy (Tan et al., 2020). Surgical resection is the most important and effective treatment method for patients with gliomas of any grade. Over the past decade, in order to achieve maximum tumor resection, various methods have been used in neurosurgery to determine functional sites, such as intraoperative ultrasound imaging, fluorescent labeling, and nerve electrophysiology functional (Sanai and Berger, 2018). Despite continuous improvement in surgery techniques, gliomas are difficult to completely remove due to their invasive growth and unclear boundaries. A variety of adjuvant therapies are used in clinical treatment. A variety of new treatment options have been reported, but they are still underdeveloped, such as electric field therapy (Stupp et al., 2017), immunotherapy (Lim et al., 2018; Huang et al., 2020; Zhang et al., 2021a), targeted therapy (Yang et al., 2022a), and nanocarrier combination therapy (Zhao et al., 2020a). Chemotherapy remains an important component of postoperative adjuvant therapy, and TMZ is the most widely used drug. In addition to GBM, in the clinical treatment of patients with recurrent or grade II–III glioma, doctors often use TMZ cautiously according to the patient’s clinical manifestations, tumor pathology, and molecular subtypes, and patient drug sensitivity must be considered.

TMZ is a synthetic small-molecule alkylating agent (Newlands et al., 1997), which can be converted into active substances at physiological pH and precisely deliver methylated fragments to DNA. Clinical trials (Hart et al., 2008; Stupp et al., 2009; Wick et al., 2012) have demonstrated a significant increase in survival and time to tumor progression in patients with GBM after intervention with TMZ. However, the objective response rate of TMZ treatment is only 4–8%, and the final outcome of most patients is still tumor recurrence and death. An important reason for the limited therapeutic effect is that patients with GBM develop resistance to TMZ during long-term standardized chemotherapy.

The cytotoxicity and mutagenicity of TMZ mainly cause DNA damage to O6-MeG (methylation of the O6 position of guanine). Regulation of O6-MeG DNA methyltransferase (MGMT) (Hegi et al., 2005; Cancer Genome Atlas Research Network, 2008; Stupp et al., 2009; Jiang et al., 2014) and an absence of DNA mismatch repair (MMR) (Higuchi et al., 2020) are now widely recognized as the most important factors contributing to TMZ resistance in gliomas. A subset of gliomas with TMZ-induced hypermutations has also been identified (Choi et al., 2018; Daniel et al., 2019). However, these discoveries have not contributed a solution to the clinical problem of TMZ resistance.

There is a growing evidence suggesting that gliomas may evolve adaptive drug resistance through TMZ-induced epigenetic changes, of which Wu et al. (Wu et al., 2021) have conducted a detailed summary. Epigenetics aims at heritable changes in gene expression—without alterations in DNA sequence—using DNA methylation, histone modification, RNA modification, chromatin remodeling, chromatin three-dimensional structural composition, and post-transcriptional regulation of non-coding RNAs, which are all mechanisms that coordinate the regulation of chromatin structure and gene expression. Previous study on epigenetic regulation in cases of glioma drug resistance have mostly focused on genes and proteins (Belter et al., 2020). However, epigenetic drugs targeting glioma DNA-modifying proteins have not achieved satisfactory results. With the development of sequencing technology, the role of RNA modification in TMZ resistance has gradually attracted attention. Finding suitable epigenetic drugs that target the post-transcriptional level may be a novel way to break this deadlock.

There are more than 170 types of biochemical modifications of RNA in living organisms (Dong and Cui, 2020a). These RNA modifications determine the fate of mRNA by affecting RNA metabolic processes such as alternative splicing, nuclear export, maintenance of stability, induction of phase separation, and regulating translation in the mRNA life cycle (Zaccara et al., 2019). RNA modifications regulate gene expression at the post-transcriptional level, participate in maintaining a unique and sophisticated gene expression profile in cells, rapidly and reversibly affect cell phenotype, and play important roles in the healthy physiology and the pathological processes of disease (Lee et al., 2020). In recent years, RNA modification has been a focus of cancer biology; the disruption of epitranscriptomic homeostasis in the physiological state of cells may be a factor initiating carcinogenesis. Moreover, the epistatic regulation of RNA is plastic and adaptive, which can induce alterations in the responsiveness of many cancers to drugs, which is one of the important reasons for the formation of acquired drug resistance in gliomas.

The regulatory roles of RNA modifications in the occurrence, development, proliferation, differentiation, invasion, and metastasis of gliomas have been mostly summarized (Dong and Cui, 2020a; Galardi et al., 2020; Dixit et al., 2021). However, the question of how RNA modifications are involved in glioma drug resistance has not been answered, and related studies are not common and remain controversial. In this review, we discuss the relationship between adaptive drug resistance and RNA modifications in gliomas and summarize the roles of several major RNA methylation modifications in the development of TMZ resistance. This work may help unearth potential drug targets and develop new epitranscriptomic drugs, which may provide clues or directions for solving the issue of glioma chemotherapy resistance and prolonging the survival of glioma patients.

## 2 The formation of acquired drug resistance in glioma

**FIGURE 1:**
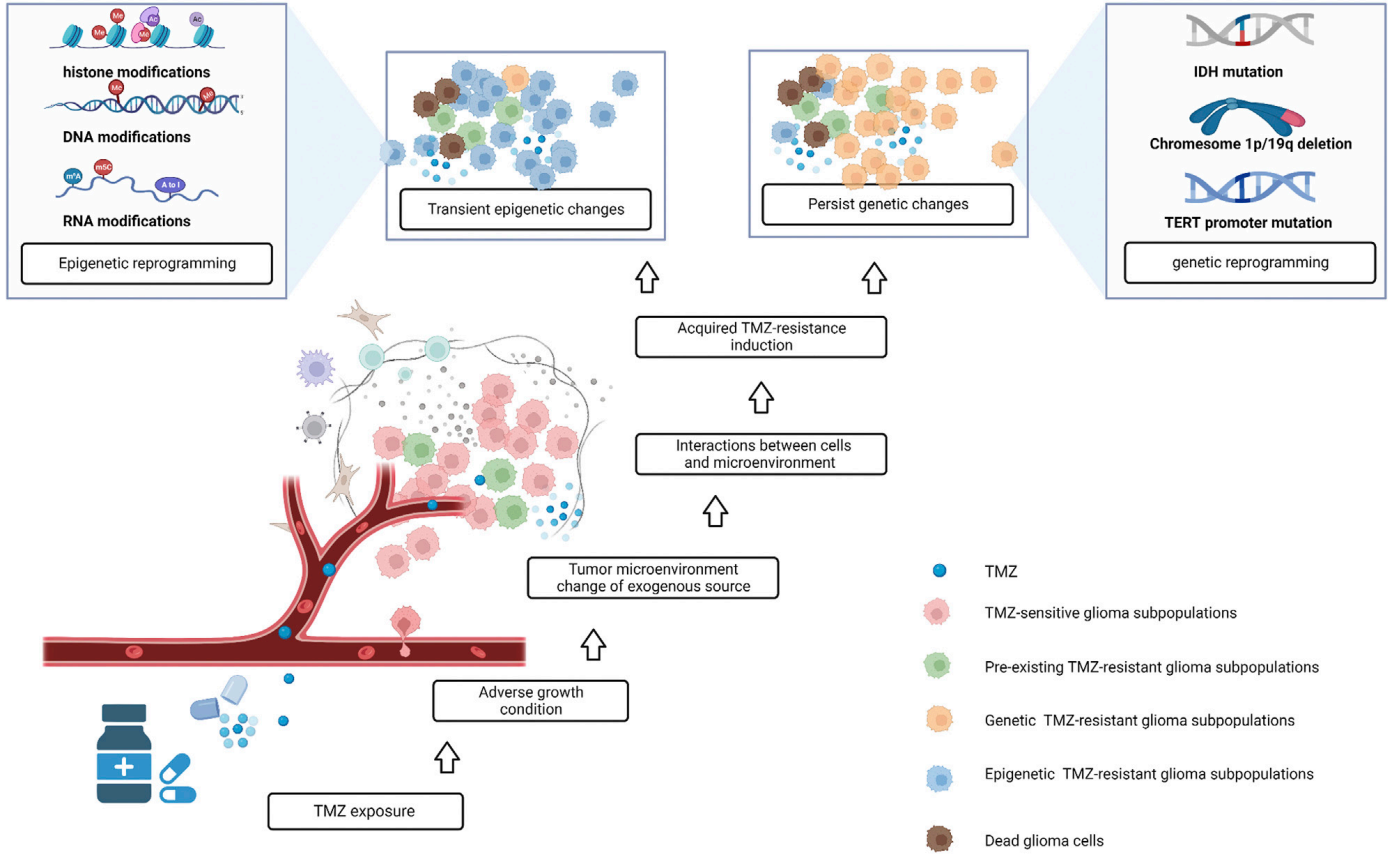
The formation of acquired drug resistance in glioma; after treatment with TMZ, some TMZ-sensitive glioma subpopulations die, and pre-existing TMZ-resistant glioma subpopulations have the potential to become dominant. At the same time, some precious TMZ-sensitive tumor cells interact with the tumor microenvironment, which has changed after TMZ exposure, and some of these cells acquire TMZ-resistance *via* epigenetic and genetic changes.

Tumor chemotherapy resistance is generally divided into primary and acquired drug resistance. Acquired resistance refers to the responses of tumors in the long-term that initially respond to treatment. Tumor heterogeneity and clonal evolution are recognized as important causes of acquired drug resistance. Glioma is highly heterogeneous; during the process of continuous proliferation and variation, tumors form many cell subclones with genomic and epigenetic diversity. The interaction between subclones and the tumor microenvironment (TME) affects the development of tumors (McGranahan and Swanton, 2017). In the past, it was believed that drug-resistant subclones existed in glioma tissue before the start of treatment. After treatment initiation, TMZ exerts selective pressure on tumor cells, and induces drug-resistant clones to become the dominant cell population, ultimately leading to drug resistance and tumor recurrence (Lee, 2016a; Garnier et al., 2018; Happold et al., 2018). Recent studies based on next-generation sequencing have emphasized the role of epigenetics, whereby some cells can survive initial drug treatment through rapid epigenetic adaptation. Signaling in the TME is transduced into cells, through DNA methylation, histone modifications, chromatin conformational changes, and RNA modifications, resulting in transient or persistent resistance phenotypes in tumors before permanent resistance emerges (Eyler et al., 2020) (Figure 1). This is a flexible and reversible phase in the therapeutic response and is the key to reversing drug resistance in gliomas.

Among glioma cell lines, A172, U87, U251, and U373 are more sensitive to TMZ, while LN-18, T98G, and U138 are somewhat TMZ-resistant, with the former having a lower IC50 (concentration required to reduce the number of surviving cells by 50%; an indicator of drug efficacy) than the latter. However, sensitive cell lines can also be induced by TMZ to acquire resistance, which to some extent mimics the presence of primary and acquired resistance to TMZ in patients (Lee, 2016b).

RNA modification can act indirectly by regulating MGMT gene expression or directly targeting TMZ-related cell death pathways, including drug metabolism and transport, drug targeting, cell death signaling, DNA damage repair, the TME, immunity responses, and maintenance and differentiation of cancer stem cells, among others (Li et al., 2020; Song et al., 2020). Almost all cells have biochemical modifications in RNA that affect RNA structure, metabolism, and function and are dynamically regulated by RNA modification regulators (Boccaletto et al., 2018). Unsupervised clustering analysis of the expression profiles of RNA modification regulators in glioma tumor samples revealed specific expression profiles with specific immunosuppressive microenvironments and mesenchymal transformation, and this class of patients is highly resistant to standard therapy and has a significant survival disadvantage (Xu et al., 2021). This indicates that RNA modification regulators affect the sensitivity of glioma cells to TMZ and directly affect the prognosis of patients with glioma.

## 3 Regulation of RNA modification in TMZ resistance in glioma

**FIGURE 2:**
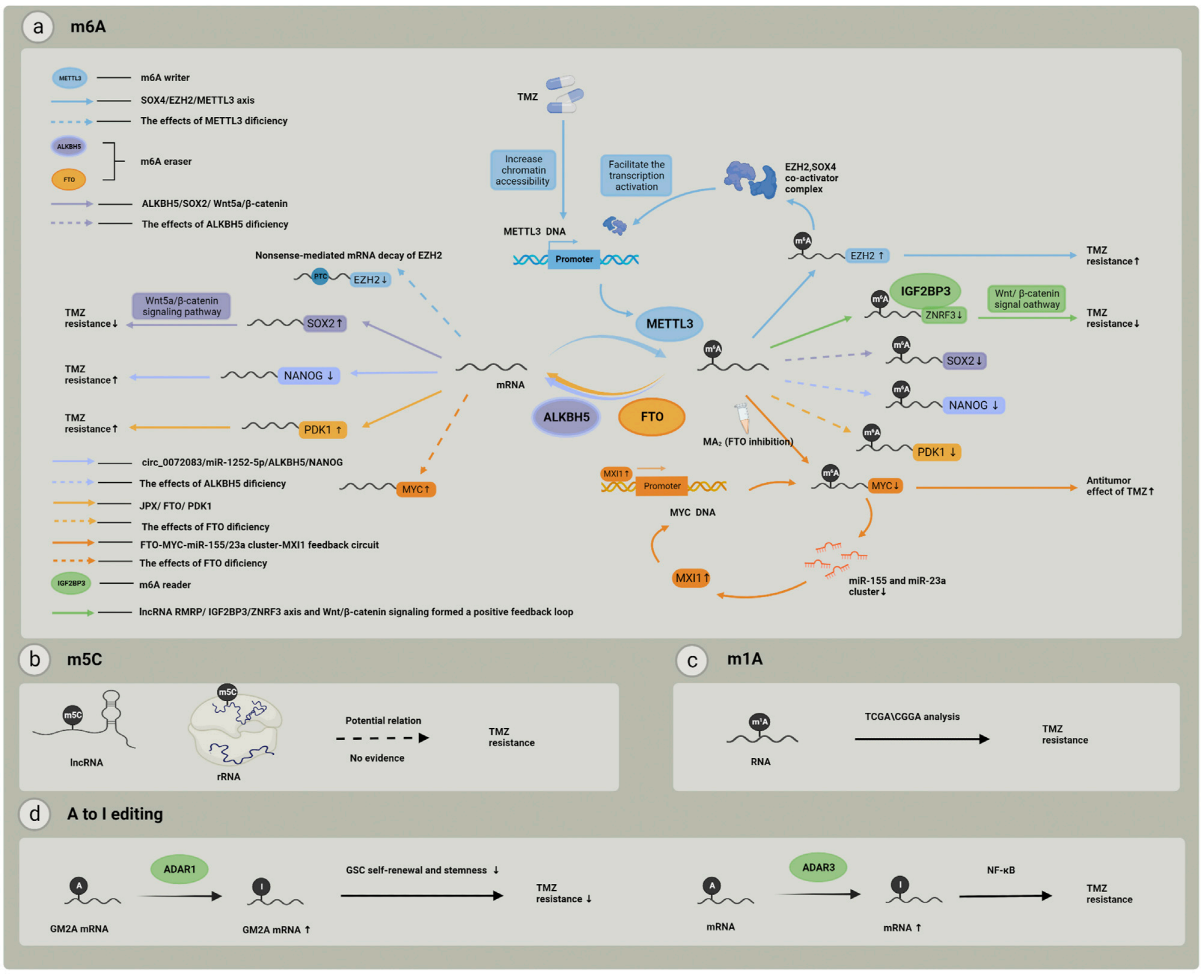
RNA modification and related regulators involved in TMZ resistance. **(A)** RNA m6A modification and its “writers”, “erasers” and “readers”, each color represents one individual pathway related to m6A modification regulators, and these pathways may finally cause different effect to TMZ treatment; **(B)** m5C modifications in rRNAs or lncRNAs may play a potential role in TMZ chemoresistance but the mechanisms still remain unclear; **(C)** RNA m1A modification related genes expression pattern has been proved to be associated with TMZ resistance; **(D)** A to I editing may effect TMZ resistance by participating in GSC self-renewal and stemness, and the important role of NF–kB pathway has also been realized.

RNA modifications mainly include N6-methyladenosine (m6A), 5-methylcytidine (m5C), N1-methyladenosine (m1A), N3-methyladenosine (m3A), N7-methylguanosine (m7G), pseudouridine, and adenosine-to-inosine (A-to-I) RNA editing. Each modification is reversible and caused by specific regulators, mainly including methyltransferases, demethylases, and methylation binding proteins. These regulators and their related mechanisms in the regulation of TMZ tolerance are provided in Table 1 and Figure 2 **.** In this section, we focus on RNA modifications of m6A and m5C that play strong roles in TMZ resistance in gliomas and tumor chemoresistance.

**TABLE 1:** The roles of RNA modification in TMZ resistance of gliomas.

RNA modification regulators	Type of enzyme	Roles in gliomas	Target and biological process	Related signal pathway	Roles in TMZ resistance	Cell lines	Refs
m6A							
METTL3	writer	Oncogene or Tumor suppressor	Enhance EZH2 mRNA stability	SOX4/EZH2/METTL3 axis	Enhance TMZ resistance	U251, U87MG	Cui et al. (2017b)
FTO	Eraser	Oncogene or Tumor suppressor	Increase the stability and the translation efficiency of MYC	MYC-miR-155/23a cluster-MXI1 feedback circuit	FTO inhibition enhanced the effect of TMZ on suppressing proliferation of glioma cells	HEK293T, U87, U251, A172	Xiao et al. (2020)
			Maintain PDK1 mRNA stability	JPX/ FTO/ PDK1	Promotes TMZ chemoresistance	U251, LN229, U87, SHG-44, LN18	Li et al. (2021b)
ALKBH5	Eraser	Tumor suppressor	Increase NANOG mRNA expression	circ_0072083/miR-1252-5p/NANOG	Enhance TMZ resistance	U251, U87, 293 T	Ding et al. (2021)
			Increase SOX2 mRNA expression	LncRNA SOX2OT/ALKBH5/SOX2/ Wnt5a/β-catenin	Inhibit TMZ resistance	GBM cell lines U87 and U251, TMZ-resistant lines, U87TR, U251TR	Liu et al. (2020)
YTHDF1	Reader	Oncogene	—	MSI1/ YTHDF1	YTHDF1 knockdown promote sensitivity of GBM cells to TMZ	DBTRG-05MG	Yarmishyn et al. (2020)
IGF2BP3	Reader	Oncogene	Reduce ZNRF3 expression and mRNA stability	lncRNA RMRP/ IGF2BP3/ZNRF3 axis and Wnt/β-catenin signaling formed a positive feedback loop	Weaken the resistance of glioma cells to TMZ	U251, TMZ-resistant U251, LN229, TMZ-resistant LN229	Liu et al. (2021a)
m5C							
—	—	—	lncRNA	—	m5C related lncRNA may change TMZ sensitivity of gliomas cell	—	Zhang et al. (2022); Rajesh et al. (2020)
NSUN5	Writer	—	rRNA	—	Stress survival adaptations (reduce TMZ sensitivity of patients)	—	Janin et al. (2019)
A-to-I editing							
ADAR1	adenosine deaminases	Oncogene	Enhance GM2A expression	TYK2/ADAR1/GM2A	impair GSC self-renewal and stemness which contribute to therapeutic resistance and relapse	—	Jiang et al. (2022)
ADAR3	adenosine deaminases	Tumor suppressor	—	ADAR3/NF-κB	increase cell survival to temozolomide	U87	Raghava Kurup et al. (2022)
m1A							
m1A regulators	—	—	—	Analysis by TCGA, CGGA	Relate to temozolomide resistance	—	Mao et al. (2022)

### 3.1 m6A

#### 3.1.1 m6A modification and its regulatory proteins

RNA m6A modification occurs in approximately 25% of mRNAs and is a dynamic and reversible process that is coordinated by the activity of methyltransferase writers (METTL3, METTL14, WTAP, and METTL16), demethylase activity erasers (FTO and ALKBH5), and m6A-modified readers (YTHDF2 -3 and YTHDC1) working in concert (Liu et al., 2018; Yang et al., 2018); multiple RNA-binding proteins are also involved in the regulation of this process. These m6A modifier proteins are frequently up- or downregulated in human cancer tissues and are often associated with poor patient outcomes (Lan et al., 2021).

The RNA methyltransferase m6A, which provides the methyl group from S-Adenosyl methionine (SAM) and transfers it to the substrate adenosine, has more than 200 members, but only a few have been identified by specific structural features and functions (Oerum et al., 2021). In mammals, especially in humans, the heterodimers formed by the RNA methyltransferases METTL3 and METTL14 are the predominant m6A RNA methyltransferases, of which WTAP and KIAA1429 are important components (Lence et al., 2016). The METTL3–METTL14 complex functions primarily in mRNA to induce adenosine methylation in a specific conserved domain (DRACH; D = A, G or U; R = A or G; H = A, C, U). This conserved domain is present in many mRNAs, but not all mRNAs are recognized by this conserved domain; only specific mRNAs can be catalyzed by METTL3 to undergo m6A methylation. The mechanisms by which this site and transcript specificity exist are unclear. The majority of methyltransferases and demethylases are fixed in the nucleus, and the process of methylation usually occurs in the nucleus in nascent mRNAs that are bound to chromatin. Transcription factors, histone modifiers, RNA binding proteins, RNA polymerases, and many other molecules can recruit m6A RNA methyltransferase and methylate newly transcribed mRNA (Zaccara et al., 2019). Other molecules are involved in RNA methylation in eukaryotes; for example, METTL16 can bind to many types of RNAs, but only some have been shown to be substrates for methylation modifications (Brown et al., 2016). METTL5:TRMT112 (van Tran et al., 2019) and ZCCHC4 (Ma et al., 2019) are important enzymes regulating rRNA methylation.

m6A-RNA pull-down experiments were the first to identify the YTH structural domain-containing RNA-binding proteins YTHDF2 and YTHDF3 (Dominissini et al., 2012). The YTH domain selectively binds the methylated portion of m6A *via* an internal tryptophan. RNA reading proteins are mainly divided into three main families, YTHDC1, YTHDC2, and YTHDF. YTHDC1 is the main nucleophile that binds rapidly to mRNA after methylation, regulates mRNA splicing by recruiting pre-mRNA splicing factors, and allows access to the target binding regions of mRNAs (Xiao et al., 2016). FTO and ALKBH5 were successively found to have demethylase activity and are both closely related to the growth and proliferation of glioblastoma stem cells (GSCs). FTO promotes the growth and self-renewal of GSCs, which is required for substantial tumor progression (Cui et al., 2017a). Overexpression of ALKBH5 has also been shown to be essential for GSC proliferation and tumorigenesis and can lead to poor prognosis (Zhang et al., 2017).

#### 3.1.2 m6A modification in glioma

Bioinformatics analysis of the China Glioma Genome Atlas Project (CGGA) microarray and RNA-sequencing database revealed that both m6A writers and readers were significantly increased, while erasers such as FTO were relatively decreased (Dong and Cui, 2020b). Xu et al. (Xu et al., 2020a) revealed that 15 m6A regulators are independent prognostic genes using Kaplan–Meier analysis. Patients with low expression of m6A regulators often have favorable prognoses, whereas patients with low expression of FTO, the eraser of m6A modification, have lower survival time compared to those with high FTO expression. These regulatory proteins are involved in the maintenance of the pathological state of gliomas and are highly related to the progression and development of gliomas; however, how m6A modification regulates TMZ resistance in glioma remains unclear.

A PubMed search for the keywords “m6A” and “drug resistance” showed a rapid increase in relevant literature in the last 3 years. This retrieved a total of 99 articles excluding irrelevant and review articles, of which only four articles were about glioma. Gastrointestinal cancers accounted for 30% of articles, lung cancer and reproductive tumors each for 22%, and leukemia 11%, and the other literature was scattered over the fields of skin cancer, bone tumors, lymphomas, thyroid cancer, and urological tumors. With another search of the “References” of these four articles and “similar articles” provided by PubMed, we found another four articles referring to RNA m6A modification in chemotherapy-resistant gliomas. These studies provide some clues to help understand the role of RNA m6A modification in glioma chemotherapy resistance. One study (Li et al., 2021a) conducted a comparative analysis of m6A modifications in TMZ-resistant and TMZ-sensitive clinical GBM samples using m6A miCLIP-seq and revealed that the m6A methyltransferase METTL3 and the EXH2 methyltransferase H3K27me3 form a regulatory loop that exacerbates resistance to TMZ in patients with GBM. The expression of METTL3 was upregulated in drug-resistant cells, resulting in increased m6A modification in the mRNA of the histone modification regulator EZH2. These modifications protected the mRNA of EZH2 from nonsense-mediated mRNA decay), and EZH2 can increase TMZ resistance in gliomas. Several additional studies have also provided other possible mechanisms. For example, a study in 2017 found that overexpression of METTL3 and treatment with the FTO inhibitor MA_2_ suppressed the growth and self-renewal of GSCs, which are related to the chemo- and radiotherapy-resistance (Cui et al., 2017b). Another study in 2020 provided further clarification about the molecular mechanisms of FTO’s influence on the reaction of glioma cells to TMZ; this may work increasing the stability and translation efficiency of Myc and then participating in regulating the MYC-miR-155/23a Cluster-MXI1 feedback circuit, which is related to the proliferation and tumorigenesis of gliomas (Xiao et al., 2020). JPX (lncRNA proximal inactive specific transcript) mediates PDK1 mRNA demethylation in glioma drug resistance by combining with the m6A demethylase FTO (Li et al., 2021b). The FTO/m6A/MYC/CEBPA signaling pathway may be related to TMZ resistance in glioma cells (Su et al., 2018). ALKBH5-mediated m6A demethylation can increase the expression of NANOG and SOX2 and promote drug resistance in gliomas (Liu et al., 2020; Ding et al., 2021). Furthermore, one study indicated that knockdown of the m6A reader YTHDF1 caused a reduction of GSCs, which may explain the elevated sensitivity of GBM cells to TMZ (Yarmishyn et al., 2020).

The number of relevant studies is too small to form a systematic map of RNA m6A modifications. In general, recent studies have demonstrated that the RNA m6A writer METTL3 can enhance the resistance of glioma cells to TMZ, and the m6A readers such as YTHDF1 and IGF2BP3 may impair this resistance. Different from the exact roles these regulators play, demethylases like FTO and ALKBH5 are both positively and negatively regulated by different cellular signaling pathways (Table 1). Even the same regulatory proteins can have multiple effects on the TMZ-resistant phenotype, and interactions among different regulators of m6A make this more complex. In addition, lncRNA (Liu et al., 2021a), miRNA, histone modification regulatory proteins, and intracellular small-molecule metabolites are also involved in the regulatory process of RNA m6A modification, which further enables RNA m6A modulation of the regulation of drug resistance in glioma.

#### 3.1.3 The effect of m6A modification on tumor chemotherapy resistance is related to tumor type

RNA m6A-modified proteins inhibit or enhance cancer cell sensitivity and resistance to cancer therapy, depending on the individual m6A-modified protein, cancer type, and specific chemotherapeutic agent. In hepatocellular carcinoma, METTL14 induced HNF3γ mRNA m6A modification, resulting in reduced HNF3γ expression and poor response to treatment with the chemotherapy agent sorafenib (Zhou et al., 2020). METTL3 mediates methylation of FOXO3 mRNA and enhances the stability of this RNA by binding to YTHDF1, while overexpression of FOXO3 led to increased sensitivity to sorafenib (Lin et al., 2020). Furthermore inhibition of METTL3-mediated m6A modification of p53 mRNA through the apoptotic pathway enhanced hepatocellular carcinoma sensitivity to sorafenib (Ke et al., 2022). In addition to mRNA m6A modification, circRNA (Xu et al., 2020b) and lncRNA (Kong et al., 2022) m6A modification in hepatocellular carcinoma can also affect drug resistance through multiple pathways. In ovarian cancer, the demethylase ALKBH5, which mediates JAK2 m6A demethylation to activate the JAK2/STAT3 signaling pathway, enhances resistance to cisplatin treatment (Nie et al., 2021a). In non-small cell lung cancer, METTL3-initiated m6A mRNA methylation increased the m6A/A ratio of YAP mRNA, promoted YAP mRNA translation by recruiting YTHDF1/3 and eIF3b, and increased YAP mRNA stability by regulating the MALAT1-miR-1914-3p-YAP axis; increases in YAP expression and activity can induce drug resistance and metastasis in non-small cell lung cancer (Jin et al., 2019). Similar m6A-mediated pathway changes have been observed in bladder cancer (Wei et al., 2021), colorectal cancer (Chen et al., 2021a), and other tumors.

After summarizing different types of tumor chemotherapy resistance mechanisms, we found that the vast majority of studies focused on m6A. The discovery of modified targets focuses on the regulatory mechanisms and downstream signaling pathways of the target. The effect of this target on tumor chemotherapy resistance was demonstrated through cell experiments and animal experiments. These studies suggested that, in drug-resistant tumor cells, RNA m6A modifications can regulate multiple signaling pathways, constituting complex epitranscriptional regulatory homeostasis. However, these studies rarely explored in depth the biological processes of tumor cell adaptation and survival in the drug environment by m6A modification targets and related signaling pathways, and the interactions between different signaling pathways and their ultimate impact on tumor cell phenotype have been rarely explored.

RNA m6A-modifying regulators are potential therapeutic targets for overcoming tumor chemotherapy resistance, and laboratories and drug researchers have begun to develop small molecule activators or inhibitors of m6A modifying proteins. A study (Yankova et al., 2021) on STM2457, an inhibitor of METTL3–METTL14 catalytic activity, screened 250,000 drug-like compounds, and their pharmacological effects were validated using *in vitro* experiments, animal leukemia models, and additional structure-based drug development approaches (Bedi et al., 2020; Moroz-Omori et al., 2021). UZH1a, a METTL3 inhibitor with good selectivity and cellular activity, has also been confirmed to significantly reduce the m6A/A ratio in mRNA. Small-molecule activators or inhibitors of METTL3, as well as inhibitors of FTO, ALKBH5, and IGF2BP1, showed considerable anticancer effects in some *in vitro* and animal models of cancer, suggesting that RNA m6A modification has good application prospects in tumor therapy (Lan et al., 2021). However, the role of epigenetic drugs targeting RNA m6A modification regulators in glioma is unknown and awaits further exploration.

### 3.2 m5C

#### 3.2.1 m5C modification and regulatory proteins

m5C is a well-known methylation modifier shown in several RNA types, and is catalyzed by NSUN protein family members containing NOL1/NOP2/SUN structural domains or DNA methyltransferase homologs (TRDMT1/DNMT2), with S-Adenosyl methionine as the methyl group donor (Agnihotri et al., 2012). m5C is relatively abundant in tRNA and rRNA, less abundant in mRNA, and relatively conserved across species. The knowledge on m5C regulatory enzymes comes mainly from studies on tRNA cytosine methylation (Trixl and Lusser, 2019a). Similar to m6A, m5C has corresponding writers (m5C methyltransferases), erasers (m5C demethylases), and readers (m5C binding proteins) that dynamically regulate intracellular m5C levels.

Ribosomal RNA in all living organisms shows m5C modifications (Motorin et al., 2010), a process mediated mainly by NSUN1 and NSUN5, while NSUN2, NSUN6, TRDMT1, and DNMT2 (Tuorto et al., 2012) have been shown to act on cytoplasmic tRNA. Studies have also found that NSUN2 has a broad range of targets and targeted RNA types (e.g., mRNA and lncRNA) (Squires et al., 2012). One study of (Yang et al., 2017) knockdowns of NSUN family member genes revealed that, among them, only NSUN2 significantly affected m5C levels in mRNA, while the study characterized the specific binding protein ALYREF using biotin-labeled oligonucleotides with or without m5C for RNA affinity chromatography and mass spectrometry. ALYREF is an mRNA export adapter, suggesting that m5C modification may facilitate the nuclear export of mRNA (Liu et al., 2021b). In addition to ALYREF, the readers of m5C include YBX1 (Chen et al., 2019) and FMRP (Yang et al., 2022b). YBX1 is beneficial for preserving the stability of target mRNA, and FMRP is associated with cellular repair and survival in cancer. m5C erasers are members of the TET family and ALKBH1, but whether these proteins act as demethylases remains to be demonstrated. These dynamic elements regulating m5C are summarized in a detailed report by Chen et al. (Chen et al., 2021b).

The methylation m5C loci in mRNA has been controversial, tied to methodological limitations and a process of continuous refinement. Squires et al. (Squires et al., 2012) first combined RNA bisulfite conversion and next-generation sequencing (RNA-BisSeq) to map over 10,000 m5C sites in 2 million cytosines of the human HeLa cell line transcriptome in 2012. In addition, a variety of immunoprecipitation-based m5C detection methods have also emerged, such as Aza-IP (Khoddami and Cairns, 2013; Khoddami and Cairns, 2014) (direct capture of RNA methyltransferase target RNA), miCLIP (Hussain et al., 2013a), m5C-RIP (using m5C monoclonal antibodies). Although these techniques can corroborate the partially deposited m5C sites in RNA, the degree of overlap between the resulting m5C profiles is low (Hussain et al., 2013b). The shortcomings of the detection methods make the sequencing results inevitably noisy; therefore, the detected m5C-related sites are controversial (Trixl and Lusser, 2019b).

A decade of technological development has updated the picture of m5C distribution in RNA, with ever-optimized bisulfite transformation protocols and more stringent analytical treatments being more often applied to m5C sequencing in different species and tissue classes. The prevailing view is now that m5C is not as widely distributed in mRNA as initially thought (Legrand et al., 2017), as shown using computational tools (Legrand et al., 2017; Huang et al., 2019a). Filtering the noise generated during bisulfite sequencing and eliminating artifacts due to incomplete deamidation or RNA secondary structures are the current directions of improvement for detecting mRNA m5C methylation sites using RNA-BisSeq. This method has confirmed that only a few of the previously identified m5C candidate sites are truly methylated marker sites, with median methylation levels of m5C sites of only 15–18%. In mammals, more than 60% of sites are hypomethylated (<20% methylation) and less than 10% are moderately or highly methylated (>40%) (Huang et al., 2019a).

#### 3.2.2 Effects of RNA m5C modification on chemotherapy resistance and glioma

Due to methodological limitations, the molecular role of m5C in mRNA and its biological links to pathophysiological processes remain unclear. Despite recent advances, a clear consensus on the distribution of m5C sites in mRNA has not been established, and reliable annotation of physiological functions is lacking (García-Vílchez et al., 2019). In non-coding RNAs, the functions of m5C modifications are relatively clear. At the molecular level, cytosine methylation modifications within RNAs can increase their structural stability, which may affect the structure and function of ribosomes and tRNA (Motorin and Helm, 2010; Bohnsack et al., 2019; Gkatza et al., 2019). The m5C methyltransferases function in a variety of cellular pathways, mainly involved in stress responses, development, and cell differentiation (García-Vílchez et al., 2019). Among them, NSUN2 is the target of the proto-oncogene *c-Myc*, which is most closely linked to tumor development (Okamoto et al., 2012) and most likely to be involved in the formation of chemotherapy drug resistance.

NSUN2 is highly expressed in lung cancer (Pan et al., 2021), gastric cancer (Hu et al., 2021), and bladder cancer (Chen et al., 2019). NSUN2 also plays an important role in chemoresistance of tumors, as inhibition of post-transcriptional RNA cytosine methylation modifications can lead to a general decrease in intracellular protein synthesis by activating stress response pathways, and this inhibition makes tumor stem cells sensitive to cytotoxic stress and unable to regenerate and proliferate under chemotherapy drug stress (Blanco et al., 2016). This idea was verified in vitro experiments, where NSUN2 deletion increased the sensitivity of tumor cells to the anticancer drugs 5-fluorouracil and cisplatin in a mouse tumor model (Blanco et al., 2016). In the HeLa cell line, combined knockdown of NSUN2 and METTL1 enhanced the sensitivity of cells to 5-fluorouracil (Okamoto et al., 2014). In esophageal cell carcinoma, NSUN2 upregulated methylated lncRNA, inhibited cisplatin-induced apoptosis, and increased chemoresistance in esophageal squamous cell carcinoma (Li et al., 2018a).

The number of studies on RNA m5C modifications in tumor drug resistance is very small, and its role in glioma drug resistance has not been reported. In glioma, tumor samples showed changes in the expression of several m5C regulators compared to normal tissue, with significant upregulation of ALYREF and NSUN3, downregulation of NSUN2-5 and DNMT1, and no significant changes in TRDMT (Zhang et al., 2022). Analysis of cancer gene mapping data revealed that differential expression of m5C methyltransferase genes in gliomas was significantly associated with clinicopathological features, malignant progression, and prognosis of gliomas. Wang et al. (2020) constructed a risk signature to predict prognosis in patients with glioma. They found that high expression of NOP2, NSUN4, NSUN5, and NSUN7 was linked to poor prognosis, whereas high expression of NSUN6 was linked to better prognosis (Li and Meng, 2021). The ability of NSUN2 to regulate cell migration by promoting mRNA cytosine methylation has been demonstrated in the brain cancer cell line U87 (Xu et al., 2020c; Wang et al., 2020). This evidence describes the role of RNA m5C modifications in the biological behavior of glioma proliferation, differentiation, migration, and malignancy, suggesting the existence of potential m5C epistatic transcriptional modalities affecting temozolomide resistance in glioma.

Similar to NSUN2, deletion of NSUN5 leads to unmethylated rRNA that can drive an overall reduction in protein synthesis, an adaptive translational program developed by cells to survive stress conditions. Epigenetic inactivation of NSUN5 is a hallmark of long-term survival in patients with glioma, but paradoxically, this adaptive regulation may also allow cancer cells to survive in the face of destructive stress conditions (chemotherapeutic agents) to avoid death and ultimately reduce patients’ therapeutic sensitivity to TMZ (Janin et al., 2019). In addition, different m6A/m5C/m1A/m7G-related lncRNA expression patterns have been reported to correlate with glioma prognosis and drug sensitivity (Shao et al., 2022). The characteristic expression patterns of m5C-related lncRNAs were associated with MGMT promoter methylation, epithelial mesenchymal transition (Zhang et al., 2022), tumor immune response, and cell cycle disruption and hypoxia (Zhou et al., 2022), and it may also alter the sensitivity of tumor cells to TMZ-induced cytotoxic effects (Rajesh et al., 2020; Zhang et al., 2022).

The above studies provide clues that RNA m5C modifications affect drug resistance in glioma, but strong direct evidence is lacking. Current research mainly focuses on the perspectives of the m5C methyltransferase NSUN family and m5C-associated lncRNA expression profiles. Maintenance and differentiation of tumor stem cells, adaptive changes in tumor cells under stressful conditions, and alterations in the tumor immune microenvironment are research directions worthy of further exploration.

### 3.3 Adenosine-to-inosine RNA editing

#### 3.3.1 A-to-I editing and its regulatory protein

A-to-I editing is one of the most abundant RNA modifications, occurring at specific sites in the structure of double-stranded RNA. Adenine nucleotides, catalyzed by the adenosine deaminase family (adenosine deaminases acting on RNA; ADARs) are deaminated to hypoxanthine nucleotides (inosine), also called inosine. During translation, the I on the mRNA is recognized by the ribosome as G (Guanosine), so A-to-I editing alters the amino acid translation codon in the protein-coding sequence, affecting the structure and function of the protein. This pairing also causes a mismatch between RNA sequences and genetic DNA sequences and is used for genomic localization (Heraud-Farlow and Walkley, 2020). Vertebrates have three ADAR genes, *ADAR1* (Kim et al., 1994), *ADAR2* (Melcher et al., 1996a) and *ADAR3* (Melcher et al., 1996b; Chen et al., 2000). ADARs have common functional domains. The dsRNA-binding domain (∼65 amino acids), which has an α-β-β-β-α configuration, makes direct contact with dsRNA (Stefl et al., 1993). The carboxyl-terminus region contains the deaminase domain that forms the catalytic center of an ADAR. In addition to the three vertebrate ADARs, testis nuclear RNA-binding protein (TENR; also known as ADAD1), which is specifically expressed in testes and is required for spermatogenesis (Schumacher et al., 1995), and TENR-like (TENRL; also known as ADAD2), which is expressed in the brain (McKee et al., 2005), have sequence and domain-structure similarity to ADAR but have no deaminase activity, owing to a lack of amino acid residues that are crucial for the catalytic reaction.

#### 3.3.2 A-to-I editing in glioma

Glutamate receptor-mediated signal transmission is associated with the pathogenesis and invasiveness of glioma. The glutamate receptor is re-edited by A-to-I editing at the glutamine (Q)/arginine (R) site of the glutamate receptor subunit B to a degree of nearly 100% in the central nervous system, which is essential for normal receptor function (Maas et al., 2001). It is widely believed that glutamate receptor subunit B mRNA is insufficiently edited in malignant gliomas. However, there is nearly no difference in the expression of ADAR2 between human glioblastoma cell lines (U87, U251, A172) and normal human glial cells (HA 1800) (Li et al., 2015). An alternative splicing variant of ADAR2 with low enzyme activity was finally proven to be the reason for this, and it is dominantly expressed in gliomas and causes the reduced RNA editing of the GluA2 subunit at the glutamine/arginine site in glioma (Wei et al., 2014; Li et al., 2015). ADAR3, a brain-specific, high-expression adenosine deaminase, has been shown to play a similar role to ADAR2 in gliomas (Oakes et al., 2017), and ADAR3 has shown great clinical significance as a potential therapeutic target and useful prognostic factor (Zhang et al., 2018) In the CGGA dataset, downregulation of ADAR3 expression correlated with glioma progression. Zhang et al. 2018) found that ADAR3 was highly expressed in neural molecular subtype glioma and cases with IDH1/2 mutation. Moreover, high expression of ADAR3 predicted a better prognosis in patients with lower-grade glioma, and multivariate analysis suggested ADAR3 expression was an independent prognostic indicator. Jiang et al. (Jiang et al., 2022) found that ADAR1 and GM2A gene that encodes G (M2) ganglioside activator protein were also elevated in GSCs compared with healthy neural stem cells, which impaired GSC self-renewal and stemness. It has further been widely demonstrated that A-to-I editing plays a role in GBM cell proliferation, migration, and invasion (Cesarini et al., 2018; Patil et al., 2020).

#### 3.3.3 The potential regulatory mechanisms of A-to-I RNA editing in temozolomide resistance

Numerous studies suggest that A-to-I RNA editing is involved in tumor chemotherapy resistance *via* multiple pathways. Studies have described relevant pathways in breast cancer (Nakano et al., 2017) and lung adenocarcinoma (Hua et al., 2022), but unfortunately there is no sufficient description for glioblastoma. A study in 2022 (Raghava Kurup et al., 2022) showed direct experimental evidence that ADAR3 expression increased temozolomide resistance in GBM cells. In that study, Kurup et al. revealed 641 genes that were differentially expressed between control cells and ADAR3-expressing cells, and most of these genes were regulated by NF-κB signaling. Further, GSCs seem to be an essential breakthrough target for A-to-I RNA editing in GBM. ADAR impair GSC self-renewal and stemness through multiple pathways and then may change the reaction to TMZ in gliomas. For example, ADAR1 binds in the 3′-UTR of GM2A for A-to-I editing and links GSC self-renewal to GM2 ganglioside catabolism. TYK2 inhibitors regulate ADAR1 *via* the upstream JAK/STAT pathway and provide a potential clinical utility (Jiang et al., 2022).

Usually, A-to-I editing acts directly in resistance-related metabolic pathways. In breast cancer, it upregulates the expression of dihydrofolate reductase (DHFR), a target of the chemotherapeutic drug methotrexate, enhancing the proliferation and chemoresistance of breast cancer cells (Nakano et al., 2017). However, the interaction between A-to-I editing and other regulators seems to make a substantial contribution in gliomas. A-to-I editing is significantly enriched in non-coding RNAs and affects the structure and function of non-coding RNAs, which have been shown to be involved in the regulation of drug resistance (Paul et al., 2017; Anthiya et al., 2018; Li et al., 2018b; Li et al., 2019a; Nie et al., 2021b; Tomar et al., 2021). A-to-I editing can also affect the expression of pharmacokinetic- and pharmacodynamic-related genes by mediating changes in miRNA structure and function that determine drug efficacy and toxicity (Nakano and Nakajima, 2018). In general, A-to-I editing and RNA m6A modification always occur independently and do not compete to bind the same sites due to differences in the sequence or structure of the acting RNAs. However, one study (Xiang et al., 2018) demonstrated that m6A-induced changes in secondary RNA structure may regulate the binding of ADAR to the target RNA, thus affecting A-to-I editing, and there may be mutual interference between the two modifications. A-to-I editing also interacts with non-coding RNAs (Liao et al., 2020).

The above studies provide clues for exploring the biological significance of A-to-I RNA editing in the drug resistance phase of glioma. Future studies could be conducted in the following directions: 1) combining sequencing and existing findings to uncover differences in A-to-I editing in chemoresistant and chemosensitive GBM cells, exploring their upstream and downstream pathways, and uncovering drug therapy related genes; 2) finding and integrating the interactions between A-to-I editing and other regulatory molecules to reveal the complete post transcriptional regulatory map of gene expression in glioma drug resistance, providing information for personalized medicine; 3) validating whether ADAR is a potential therapeutic target for glioma drug resistance, which can be used for adjuvant therapy after TMZ treatment by developing molecules that regulate ADAR expression or activity; 4) verifying whether A-to-I editing in glioma affects the TMZ effects by influencing genes related to alkylated drug metabolism, which may provide new options for the treatment of patients with GBM by adjusting the route of administration or co-administration of metabolic enzyme inhibitors.

### 3.4 Other RNA modifications and small-molecule regulators

Mao et al. (2022) constructed a signature based on m1A regulators with The Chinese Glioma Genome Atlas (CGGA) and The Cancer Genome Atlas (TCGA) datasets, which divided gliomas into different subtypes. Four m1A modification-related patterns were found, with different prognoses. Some characteristics of glioma such as stemness, genomic heterogeneity, TME, and immune cell infiltration were also significantly different between the poor and best prognosis clusters. One of these differentially expressed genes, ABCC3, was related to glioma proliferation and TMZ resistance, which may provide new insights into the function and potential therapeutic role of m1A in glioma. Another study showed the potential role of m7G in gliomas. Chen et al. (2022) found that 17 out of 31 regulators of m7G RNA methylation showed significantly higher expression levels in gliomas. Based on these 17 genes, they developed a risk model involving three m7G methylation regulators. Patients were divided into high- and low-risk groups. Overall survival was significantly lower in the high-risk group than in the low-risk group. This finding provides a new insight into the underlying mechanisms of m7G modification in tumorigenesis and the development of gliomas. However, there is no experimental evidence to support these analyses.

The role of other RNA modifications in GBM resistance has not been well described, but there have been some advances in the progression of GBM occurrence and clinical characteristics of patients with GBM. For example, the enzyme pseudouridine synthase 7 is associated with prognosis in patients with GBM (Hu et al., 2020; Wang et al., 2021b) and is involved in regulating the tumor immune microenvironment of GBM, promoting glioma genesis and growth (Cui et al., 2021). Small-molecule inhibitors targeting pseudouridine synthase 7 prolong the life span of mice with GBM (Zhang et al., 2021b). Dysregulation of m1A regulators is closely related to glioma development and progression, and inhibition of TRMT6, a methyltransferase of m1A, inhibits glioma cell proliferation, migration, and invasion (Wang et al., 2021c). These studies provide the basis for further study on the association between RNA modification and TMZ resistance.

We have also noticed the essential effects of non-coding RNA in post-transcriptional gene regulation in TMZ resistance. As a typical example, miRNA, small non-coding RNAs consisting of 20–24 nucleotides, can change the expression profiles of glioma cells through binding with target mRNAs and causing instability and translation inhibition (Zhang et al., 2010; Ahir and Lakka, 2019). miRNA directly targets TMZ resistance-related signaling pathways or interacts with other regulators indirectly. miR-26a reversed the tumor suppressive role of transcription factor AP-2α, which may enhance TMZ resistance by decreasing the expression of MGMT and programmed death-ligand 1 (Huang et al., 2019b). miR-198 enhanced TMZ sensitivity by reducing levels of MGMT and lncRNAs (H19 and HOXD-AS2), and upregulation by TGF-β1 reversed this phenomenon (Nie et al., 2021b). Overexpression of miR-519a enhanced TMZ sensitivity in glioma by inhibiting the STAT3/Bcl-2 signaling pathways (Li et al., 2018b).

Zhao et al. (2020b) found differential expression of mRNAs, lncRNAs, and circRNAs between TMZ-resistant GBMs and primary GBM tissues. They revealed a complex interaction among regulators by constructing a network of lncRNAs, mRNAs, and circRNAs. In fact, lncRNAs often influence regulation by interacting with miRNA-related pathways or, in other words, blocking them. For example, highly expressed in TMZ-resistant glioma cells, lncRNA SNHG12 completely binds with miR-129-5p, causes the upregulation of MAPK1 and E2F7, and then affects TMZ-induced apoptosis *via* the MAPK/ERK pathway (Lu et al., 2020). lncRNA SNHG15 enhances TMZ-resistance in glioma by decreasing the expression of miR-627-5p (Li et al., 2019b).

## 4 Discussion

Over the past three decades, the relative survival rate for malignancies has increased from 23% to 36%, but GBM has seen only a slight increase of 3% (Miller et al., 2021). TMZ, the most widely used and studied chemotherapeutic agent for GBM in clinical practice, has shown improvements in patient survival. However, in long-term standardized chemotherapy, patients have developed resistance to TMZ, which severely limits their survival and is an important reason for the maintenance of the 5-year survival rate in GBM. Therefore, expanding our understanding of the molecular mechanisms of drug resistance in glioma, finding new therapeutic targets to improve treatment regimens for patients with GBM, and regaining TMZ sensitivity in patients are the current focus of our work.

Advances in methodology have led to a better understanding of the regulation of gene expression in glioma drug resistance, and the involvement of RNA modification in the formation of a rapid drug resistance phenotype in glioma is an important finding of recent scientific work. Although epigenetic drugs targeting epigenomic regulators and protein modification-regulated enzymes have won approval as a new type of antitumor drugs, with the first clinical applications, there have been no major satisfactory results (Dawson and Kouzarides, 2012; Park and Turcan, 2019; Uddin et al., 2022). The reversibility of RNA modifications provides a new breakthrough to overcome the challenge of drug resistance in glioma. Studies on chemotherapy resistance in a variety of malignancies have shown that modulators of RNA modification are potential targets for drug therapy. However, the question of how RNA modifications are involved in the formation of GBM drug resistance has not been clearly answered, and there are few relevant studies. Future studies should search more for the biological significance of RNA modifications in TMZ tolerance and uncover relevant drug therapeutic targets, which may provide a theoretical basis for the development of epigenetic drugs targeting RNA modifications.

The interactions between RNA modification regulators and interactions of RNA modification with non-coding RNA regulation, epigenomics, proteomics, and metabolomics, also deserve attention. These different levels or dimensions of regulatory factors constitute a complex and stable network of gene expression regulation and are often responsible for the suboptimal clinical outcomes of new drugs. Mapping the complete epigenetic map of gene expression in glioma chemoresistance may provide clues for developing rational drug treatment regimens. Moreover, applying the detection of RNA modifying regulatory factors to the molecular pathological diagnosis of clinical patients and developing individualized treatment plans to achieve precision medicine is also a possible future development direction.
